# Supplementary figures and images for: Fatal Lassa fever in cynomolgus monkeys is associated with systemic viral dissemination and inflammation
Source: PLoS Pathog. 2024 Dec 9;20(12):e1012768. doi: 10.1371/journal.ppat.1012768 (PMC11658700; doi:10.1371/journal.ppat.1012768)

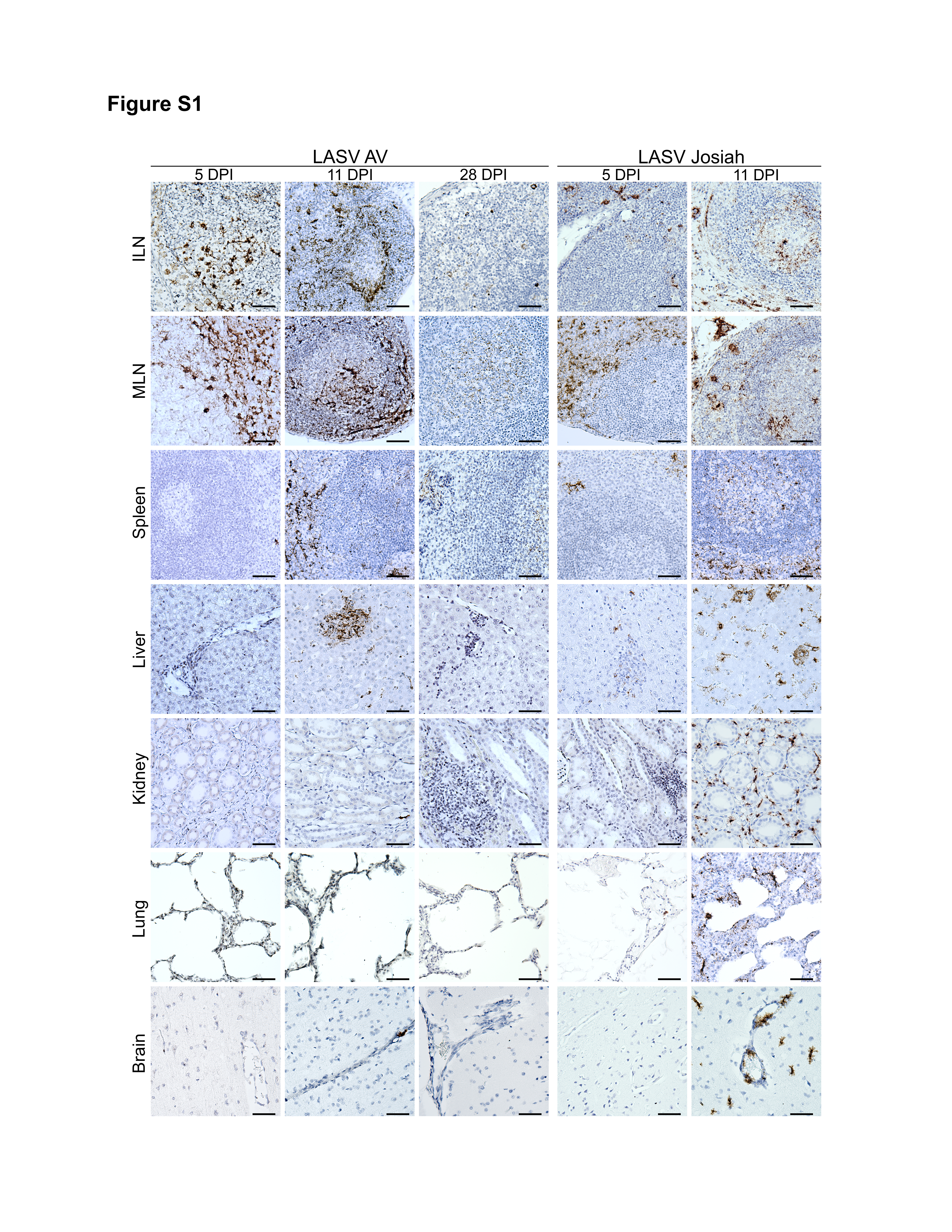

Supplement: S1 Fig — LASV RNA was detected in the various organs by ISH with AV- and Josiah-specific probes (brown). Hematoxylin staining is shown in blue. Samples obtained at 5 and 11 DPI from AV- infected and Josiah-infected animals were analyzed, together with samples obtained at 28 DPI for AV-infected animals. Scale bars: 100 μm. (TIF) [file ppat.1012768.s001.tif]

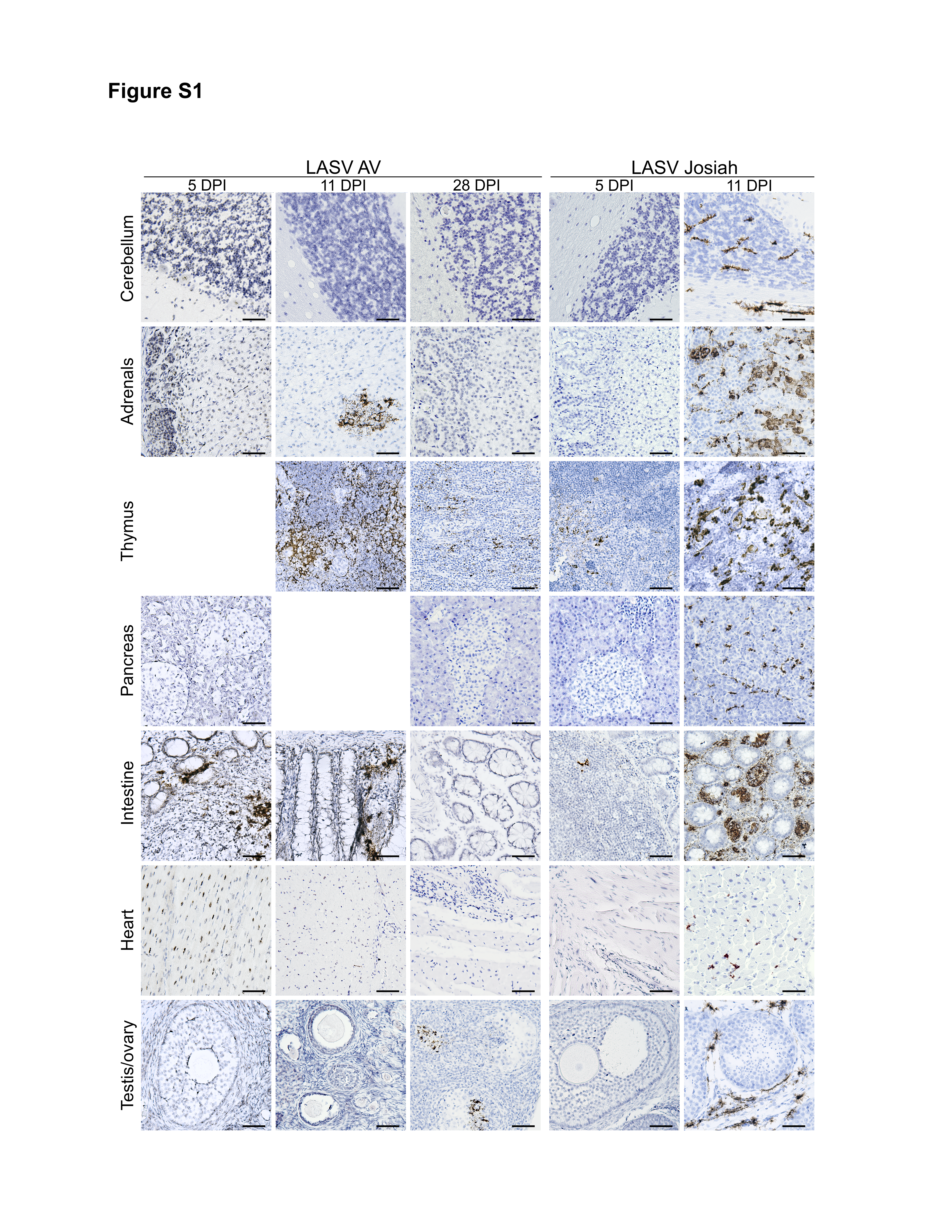

Supplement: S2 Fig — LASV RNA was detected in the various organs by ISH with AV- and Josiah-specific probes (brown). Hematoxylin staining is shown in blue. Samples obtained at 5 and 11 DPI from AV- infected and Josiah-infected animals were analyzed, together with samples obtained at 28 DPI for AV-infected animals. Scale bars: 100 μm. (TIF) [file ppat.1012768.s002.tif]

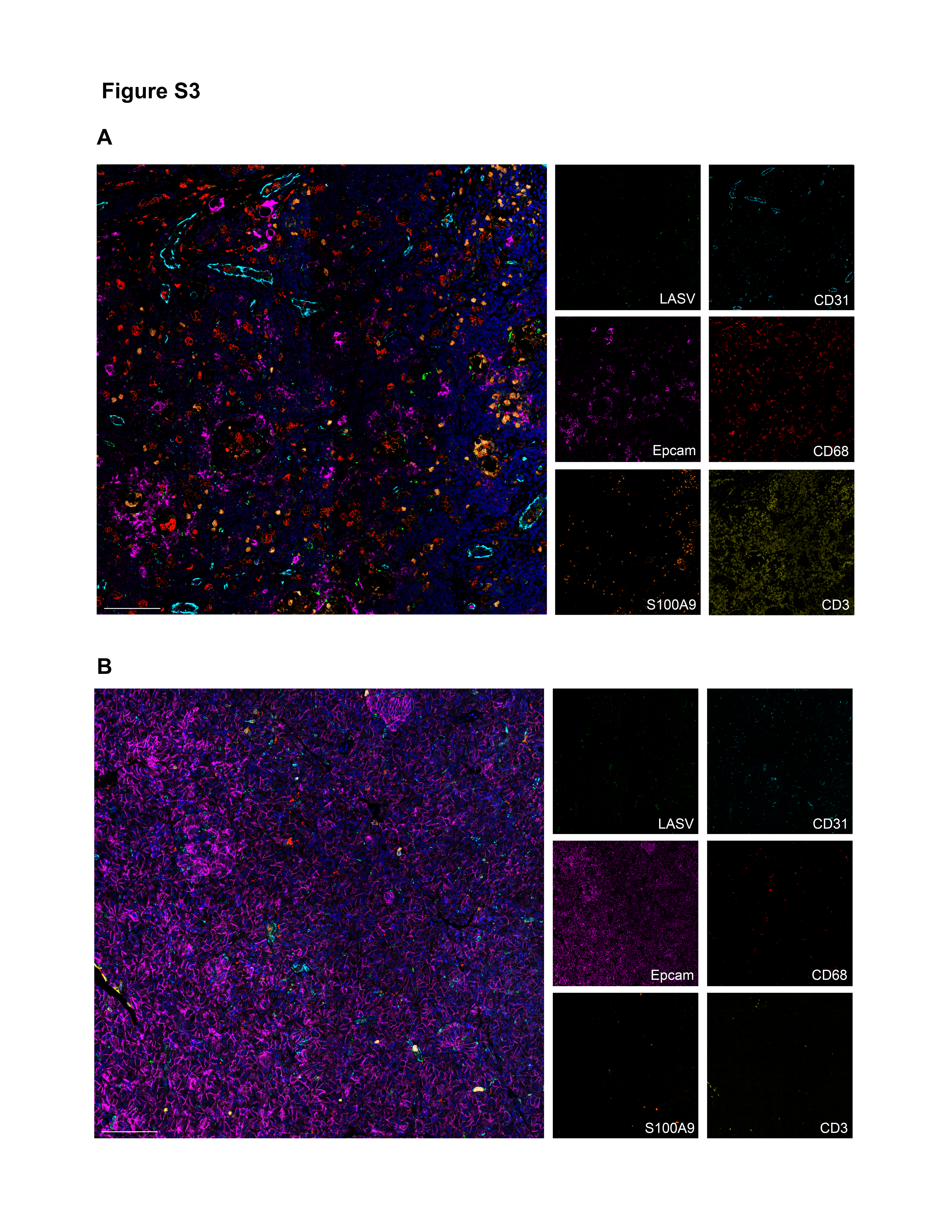

Supplement: S3 Fig — Thymus (A) and pancreas (B) sections obtained at 11 DPI from Josiah-infected animals were stained for LASV GPC (green), calprotectin (orange), CD68 (red), CD3 (yellow, not shown for the merge image of thymus), desmin (magenta), and with DAPI (blue) and analyzed by confocal microscopy. Scale bars: 100 μm. (TIF) [file ppat.1012768.s003.tif]

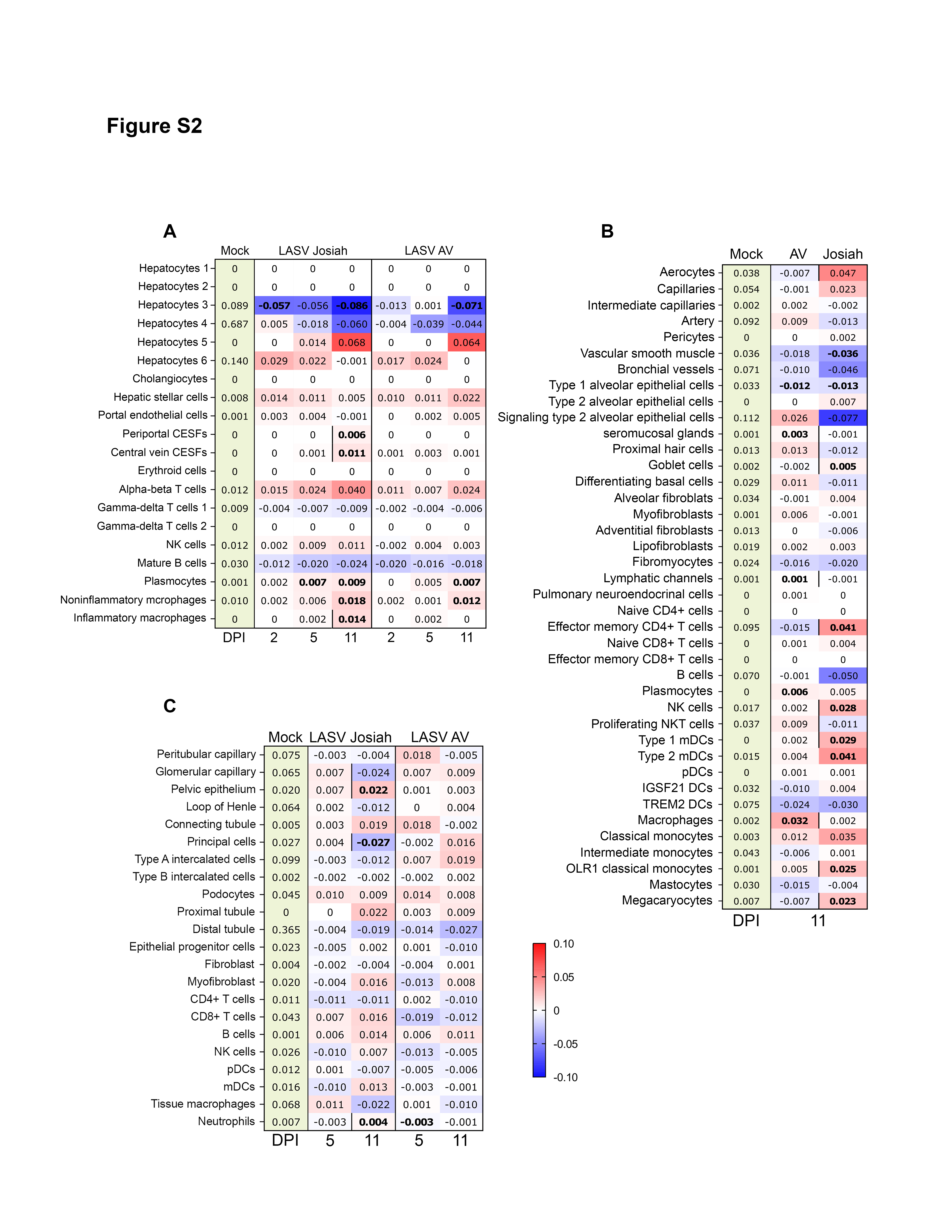

Supplement: S4 Fig — RNA-seq data obtained for the liver (A), lungs (B), and kidneys (C) of mock-infected animals or LASV-infected animals at various time points after infection were used for a deconvolution analysis by the CIBERSORT method with a matrix signature appropriate to each tissue: Baderlab for liver [liver], Krasnowlab for lungs [lungs] and kidneycell atlas for kidneys [kidneys] (n = 3 for each group). The values in the “mock” column indicate the mean proportion of each cell type within the total cell population. The values indicated in the other columns indicate the differences between the mean value for the group concerned and that for the mock-infected animals. These differences are also illustrated with the colorscale in a heatmap. Significant differences (p < 0.05) with respect to mock-infected animals are indicated by numbers in bold type, and differences between Josiah-infected and AV-infected animals at the same time point are indicated by a vertical black line. (TIF) [file ppat.1012768.s004.tif]
